# The multiple positive effects of Honghua Ruyi Pills combined with estradiol valerate and dydrogesterone tablets on postoperative recovery in women after artificial abortion

**DOI:** 10.3389/fmed.2026.1778706

**Published:** 2026-04-22

**Authors:** Bing Zhou, Jingjing Li, Yanhua Fu, Yifang Hu, Tingting Fei

**Affiliations:** Department of Gynecology and Obstetrics, Ningbo Beilun Third People’s Hospital, Ningbo, Zhejiang, China

**Keywords:** artificial abortion, dydrogesterone, endometrial repair, estradiol valerate, Honghua Ruyi Pills

## Abstract

**Objectives:**

To evaluate the efficacy of Honghua Ruyi Pills combined with estradiol valerate and dydrogesterone tablets in promoting endometrial repair and menstrual recovery after artificial abortion.

**Methods:**

This was a randomized, double-blind, double-dummy, controlled trial conducted at a single center. A total of 380 patients who underwent artificial abortion were randomly assigned to Honghua Ruyi Pills alone (Group 1, *n* = 129), estradiol valerate plus dydrogesterone tablets alone (Group 2, *n* = 118), or the combination of all three medications (Group 3, *n* = 133). The primary outcome was endometrial thickness at 1 week post-surgery. Secondary outcomes included time to postoperative abdominal pain resolution, postoperative vaginal bleeding duration, time to menstruation resumption, postoperative menstrual duration, menstrual volume, menstrual blood color, and the incidence of adverse reactions. All outcomes were assessed after a 21-day treatment period.

**Results:**

The primary outcome analysis showed that Group 3 had significantly greater endometrial thickness at 1 week post-surgery compared with Groups 1 and 2 (*p* = 0.002). For secondary outcomes, Group 3 demonstrated shorter postoperative abdominal pain duration (*p* < 0.001), bleeding duration (*p* < 0.001), time to menstrual resumption (*p* < 0.001), and menstrual duration (*p* < 0.001) compared with the other groups. Kaplan–Meier analysis indicated that patients in Group 3 achieved favorable postoperative outcomes more rapidly (all *p* < 0.001). The incidence of adverse reactions did not differ significantly among the three groups.

**Conclusion:**

In this single-center trial, the combination of Honghua Ruyi Pills with estradiol valerate and dydrogesterone tablets showed preliminary superiority in promoting short-term endometrial repair and menstrual recovery after artificial abortion compared with either treatment alone. These findings are hypothesis-generating and warrant confirmation in multicenter trials with longer follow-up and clinically relevant reproductive endpoints such as pregnancy rate and intrauterine adhesion formation.

## 1 Introduction

Artificial abortion procedures cause varying degrees of damage to the endometrium. These procedures include suction curettage, forceps dilation and curettage, mid-term induction and evacuation, and incomplete abortion evacuation, all of which can cause damage to the basal layer of the endometrium through surgical intervention in the uterine cavity (1). This damage may lead to the proliferation of fibrous connective tissue and impaired endometrial regeneration, resulting in defective endometrial repair or even the formation of intrauterine adhesions, which severely affect embryo implantation and cause secondary infertility (2–4). Patients often experience symptoms such as dysmenorrhea, reduced menstrual flow, and even amenorrhea (5, 6). In cases of missed abortion, the developmentally arrested pregnancy tissue undergoes hemorrhage and organization, adhering tightly to the uterine wall, making curettage more difficult and sometimes requiring repeated procedures. This further exacerbates endometrial damage, significantly increasing the risk of intrauterine adhesions and secondary infertility (7). The more frequently a woman undergoes artificial abortion, the shorter the interval between procedures, and the later the gestational age, the greater the risk of endometrial damage. Studies have shown that the incidence of thin endometrium after a single abortion is 20%, while it rises to 53.33% after two or more abortions (8).

Although medical abortion does not involve uterine cavity manipulation, the prolonged bleeding and the risk of secondary infections can still cause endometrial damage and repair disorders, potentially leading to intrauterine adhesions. Suppose a medical abortion is incomplete and requires curettage. In that case, the pregnancy remnants may become tightly adherent to the endometrium, especially when the pregnancy tissue undergoes organization, and repeated curettage may worsen the endometrial damage. Therefore, for patients with endometrial damage after artificial abortion, it is recommended to undergo endometrial repair treatment to promote endometrial growth and wound healing and reduce the risks of endometrial repair failure and intrauterine adhesions after abortion (9).

Currently, clinical methods to promote endometrial repair include estrogen-progestin medications, combined oral contraceptives, traditional Chinese medicine, and bioelectrical stimulation (9). Estrogen plays a central role in endometrial repair by stimulating the proliferation of endometrial epithelial cells, promoting glandular growth and angiogenesis. Progesterone, in turn, further facilitates the secretory transformation of the endometrium and enhances endometrial receptivity (10). Studies have shown that the timely supplementation of estrogen and progesterone after abortion can, to some extent, promote endometrial regeneration, increase endometrial thickness, and reduce the incidence of intrauterine adhesions (11). A retrospective longitudinal cohort study also confirmed that estrogen therapy after dilation and curettage for missed abortion can improve endometrial recovery and contribute to better long-term pregnancy outcomes (12). Oral medications are a more cost-effective and convenient treatment approach in clinical practice, with high patient compliance, minimal impact on daily life, and high satisfaction. After treatment with estradiol valerate and dydrogesterone tablets, significant improvement in intrauterine adhesions and a marked increase in endometrial thickness have been reported in women post-abortion (13). However, hormone therapy alone still presents certain limitations in clinical practice. For patients with severe damage to the endometrial basal layer, even with adequate estrogen treatment, the improvement in endometrial thickness remains suboptimal, and some patients with thin endometrium exhibit resistance to estrogen stimulation (14). Furthermore, there is currently no internationally unified standard regarding the dosage, route of administration, or duration of estrogen and progesterone therapy following abortion (15). Therefore, exploring the combination of hormone therapy with other agents to enhance endometrial repair holds profound clinical significance.

Honghua Ruyi Pills are a traditional Tibetan medicine formula with effects such as wind-dispelling, pain relief, menstrual regulation, and bruise reduction, commonly used in the treatment of gynecological diseases. Several animal and clinical studies have shown that Honghua Ruyi Pills effectively improve symptoms of primary dysmenorrhea and secondary dysmenorrhea caused by endometriosis (16–19). Regarding the safety profile in human applications, a randomized, vehicle-controlled clinical trial in patients with pelvic inflammatory disease demonstrated that the incidence of adverse reactions in the group receiving Honghua Ruyi Pills combined with antibiotic therapy was only 7.04% (5/71), which was lower than the 10.61% (7/66) observed in the vehicle control group, with no serious adverse reactions reported (20). Furthermore, safety is designated as a key monitoring endpoint in a multicenter, randomized, double-blind, vehicle-controlled clinical trial protocol investigating Honghua Ruyi Pills for the treatment of endometrioma-associated dysmenorrhea, with comprehensive safety observations conducted throughout the entire treatment period (21). As a commercially available Tibetan medicinal preparation included in the National Reimbursement Drug List, Honghua Ruyi Pills have accumulated substantial long-term clinical safety experience in the field of gynecology (22). However, the effect of Honghua Ruyi Pills on endometrial repair after artificial abortion and other related repairs remains unclear. Therefore, this study aims to explore this issue and systematically evaluate the therapeutic effects of Honghua Ruyi Pills on this patient group.

## 2 Materials and methods

### 2.1 Study participants

A total of 380 patients who underwent artificial abortion at our hospital between January 2024 and January 2025 were included in this study. These patients were both outpatient and inpatient cases, all patients signed informed consent forms and voluntarily participated in the study. All participants were aged 18–46 years old and randomly assigned to three groups. The Honghua Ruyi Pills treatment group (Group 1) included 129 patients, with a median age of 33 (28, 37) years. The estradiol valerate + dydrogesterone tablets treatment group (Group 2) consisted of 118 patients, with a median age of 33 (28, 37) years. The Honghua Ruyi Pills + estradiol valerate + dydrogesterone tablets treatment group (Group 3) comprised 133 patients, with a median age of 33 (28, 36) years.

### 2.2 Inclusion criteria

The four inclusion criteria are as follows: (1) Diagnosed as high-risk individuals with endometrial damage following artificial abortion. Specifically, patients who have undergone artificial abortion and are complicated by one or more of the following conditions: ① History of recurrent miscarriage: ≥2 previous miscarriages, missed abortion, infectious abortion, incomplete abortion with curettage, or history of placenta accreta. ② History of intrauterine surgery. ③ History of uterine infection. (2) Follow-up examination 1 week after the abortion showing no residual tissue in the uterus. (3) Participants must be aged 18–50 years old, and have regular menstrual cycles of 28–35 days. (4) The participants provided informed consent, volunteered for the study, and the process complied with the Good Clinical Practice regulations.

### 2.3 Exclusion criteria

The eight exclusion criteria of this study are as follows: (1) Allergic to the components of the medication. (2) Women who are breastfeeding or unable to ensure contraception during the trial period. (3) Clinical laboratory abnormalities: serum Ca-125 ≥35 U/mL, or erythrocyte sedimentation rate >25 mm/h. (4) Co-existing severe primary diseases such as cardiovascular, liver, kidney, and hematologic disorders, or gynecological diseases like ovarian cysts that may affect the experimental results. (5) Mental illnesses, lack of self-control, or inability to express oneself clearly. (6) Heavy smokers (>25 cigarettes/day) or chronic alcohol users (weekly intake >70 g). (7) Participation in other clinical trials within the past 3 months, or use of similar drugs or related treatments within the past 2 weeks. (8) Other situations deemed inappropriate for participation in the clinical trial by the investigators.

### 2.4 Study medications

#### 2.4.1 Honghua Ruyi Pills

Honghua Ruyi Pills are a traditional Tibetan medicine preparation officially approved by the National Medical Products Administration (NMPA) of China for clinical use (Approval No. Guoyaozhunzi Z20027000; National Standard No. WS-11268(ZD-1268)-2002). The drug is listed in the National Health Insurance Drug Catalogue of China and classified as a prescription medication. Honghua Ruyi Pills are manufactured by Gannan Foge Tibetan Pharmaceutical Co., Ltd., Gansu Province, China.

Honghua Ruyi Pills consist of 26 ingredients: *Carthamus tinctorius* (Safflower), *Crocus sativus* (Saffron), *Sinopodophyllum hexandrum*, *Terminalia chebula*, *Rubia wallichiana*, *Cinnamomum cassia*, *Corydalis hendersonii*, *Aucklandia costus*, *Coriandrum sativum*, *Dalbergia odorifera*, Cinnabaris, Fel Ursi, *Arnebia euchroma*, Halite, *Mirabilis himalaica*, *Gentiana urnula*, *Piper nigrum*, *Zaocys dhumnades* (detoxified), *Corydalis cashmeriana*, *Phyllanthus emblica*, *Hippophae rhamnoides* extract, Sal ammoniac, Lacca sinica, *Lycium barbarum*, *Aquilaria sinensis*, and Potassium nitrate. The primary pharmacological actions include promoting blood circulation, removing blood stasis, relieving pain, and regulating menstruation. The active components—safflower and saffron—have demonstrated anti-inflammatory, analgesic, and circulation-promoting properties, while cinnamon provides warming and vasodilatory effects. The synergistic action of these components is believed to facilitate uterine involution, improve local blood supply, and promote endometrial recovery after surgical procedures.

Regarding safety, the preparation contains cinnabar (a mercury-bearing mineral); accordingly, long-term use is not recommended. Known contraindications include hepatic/renal insufficiency, hematopoietic disorders, pregnancy, and lactation. Occasional adverse reactions reported in clinical use include mild allergic skin reactions, gastrointestinal discomfort, and rare respiratory symptoms. In the present study, Honghua Ruyi Pills were administered for 21 days, within the established safe duration for short-term use. Each pill weighs 0.2 g (10 pills = 2 g); the approved dosage is 1–2 g orally, twice daily. In this trial, patients received 5 pills (1 g) per dose, twice daily (daily total: 2 g).

#### 2.4.2 Estradiol valerate and dydrogesterone

Estradiol valerate is an estradiol ester that serves as a prodrug of 17β-estradiol, a natural and bioidentical human estrogen. Upon oral administration, it is rapidly hydrolyzed to estradiol, which promotes endometrial proliferation and repair. It was manufactured by Zhejiang Xianju Pharmaceutical Co., Ltd.

Dydrogesterone is a retro-isomer of natural progesterone with selective progestogenic activity. Crucially, dydrogesterone does not inhibit ovulation and does not suppress gonadotropin (luteinizing hormone and follicle-stimulating hormone) secretion at clinical dosages. It is devoid of androgenic, estrogenic, and glucocorticoid activities. This distinguishes the estradiol valerate and dydrogesterone combination from ethinyl estradiol-containing combined oral contraceptives (ethinyl estradiol/medroxyprogesterone acetate, ethinyl estradiol/drospirenone), which suppress the hypothalamic–pituitary-ovarian axis and inhibit ovulation. The estradiol valerate/dydrogesterone sequential regimen used in the present study constitutes a hormone replacement therapy, not a contraceptive regimen. Its purpose is to provide exogenous estrogen-progestogen supplementation to support endometrial recovery: estrogen promotes endometrial proliferation, while sequential dydrogesterone induces secretory transformation, mimicking the physiological luteal phase. Compared to ethinyl estradiol-containing preparations, the estradiol valerate/dydrogesterone regimen carries a substantially lower risk of venous thromboembolism, as estradiol valerate has less hepatic impact on coagulation factors than synthetic ethinyl estradiol. Dydrogesterone was manufactured by Yangzhou Aoruite Pharmaceutical Co., Ltd.

### 2.5 Randomization, blinding and treatment methods

This study was a randomized, double-blind, double-dummy, controlled trial. A computer-generated block randomization sequence (block size of 6) was created by an independent statistician using SAS software prior to patient enrollment. Participants meeting the inclusion criteria were allocated to three groups in a 1:1:1 ratio. The randomization sequence was concealed using sequentially numbered, opaque, sealed envelopes prepared by the independent statistician. Each envelope was opened only after the participant had been formally enrolled and the baseline assessment completed. The randomization list was held exclusively by the independent statistician and was inaccessible to the investigators, treating clinicians, and participants throughout the trial.

A double-dummy, double-blind design was employed to ensure adequate blinding. Matched placebos identical in appearance, shape, color, weight, and packaging were manufactured for each active medication (Honghua Ruyi Pills, estradiol valerate tablets, and dydrogesterone tablets). All participants, regardless of group assignment, followed the same dosing schedule and took the same number of tablets at the same frequency. The specific dosing regimen for each group was as follows:

Group 1 (Chinese herbal medicine group): Participants received active Honghua Ruyi Pills (2 times daily, 5 pills per dose) + estradiol valerate placebo (once daily, 1 tablet per dose) + dydrogesterone placebo (added from day 11, 2 times daily, 1 tablet per dose). Group 2 (Hormonal therapy group): Participants received Honghua Ruyi Pills placebo (2 times daily, 5 pills per dose) + active estradiol valerate (once daily, 1 mg per dose) + active dydrogesterone (added from day 11, 2 times daily, 10 mg per dose). Group 3 (Combination group): Participants received active Honghua Ruyi Pills (2 times daily, 5 pills per dose) + active estradiol valerate (once daily, 1 mg per dose) + active dydrogesterone (added from day 11, 2 times daily, 10 mg per dose). Blinding was maintained for participants, treating clinicians, and outcome assessors throughout the entire trial period. Unblinding was performed only after database lock and completion of the statistical analysis, in accordance with the pre-specified protocol.

### 2.6 Criteria for exclusion, withdrawal, and termination of the trial

During the trial, the following conditions will lead to exclusion or termination of participation: (1) Cases not meeting inclusion criteria will be excluded. (2) Patients who experience allergic reactions or severe adverse events during the trial, have poor compliance, or fail to follow the prescribed medication regimen will have their participation terminated. (3) Patients who develop severe complications after inclusion, or experience special physiological changes that make them unsuitable for continued participation, or who voluntarily withdraw from the trial, will be considered as withdrawn cases.

### 2.7 Observational indicators

The primary outcome was endometrial thickness at 1 week post-surgery, measured by transvaginal ultrasonography (in mm). This was selected as the primary endpoint because endometrial thickness is a well-established surrogate marker for endometrial recovery and is directly relevant to the therapeutic objective of promoting endometrial repair (23). Secondary outcomes included the following six indicators: (1) Time to postoperative abdominal pain resolution: defined as the time from surgery cessation to the first documented absence of patient-reported abdominal pain (Visual Analog Scale ≤1). (2) Postoperative vaginal bleeding duration: the total number of days from the day of the abortion surgery until vaginal bleeding completely ceases. (3) Time to menstruation resumption: the total number of days from the first day after the abortion surgery until the day before the patient’s next menstrual period begins. (4) Postoperative menstruation duration: the total number of days from the first day of the menstrual period to the day the period completely ends. (5) Menstrual flow after menstruation recovery: assessed using the weighed sanitary pad method, with all used pads weighed and net menstrual blood loss estimated after subtracting the dry-pad weight; values were then categorized into three levels: heavy, medium, and light (24). (6) Menstrual blood color: scored using a pictorial color assessment card with predefined scoring criteria and classified into three categories: dark reddish-brown, black-red, and red (25). In addition, the trial monitored adverse symptoms associated with the medications, including postoperative complications, gastrointestinal discomfort, breast tenderness, headaches, diarrhea, intrauterine adhesions, uterine residue, pelvic inflammation, and cyclical abdominal pain.

### 2.8 Statistical analysis

The data analysis for this study was conducted using SPSS 26.0 software. Baseline characteristics and postoperative observation indicators were first tested for normality using the Shapiro–Wilk test and for homogeneity of variance using Levene’s test. As the continuous outcome data exhibited non-normal distributions and were presented as median (25th, 75th percentiles), the Kruskal–Wallis *H* test, a rank-based non-parametric method appropriate for non-normally distributed data, was used for comparisons among the three groups. Where significant differences were detected, pairwise *post-hoc* comparisons were performed using the Dunn–Bonferroni method, with statistical significance set at a Bonferroni-adjusted threshold of *p* < 0.0167 (i.e., *p* < 0.05/3). Categorical data and adverse symptoms were presented as n (%), and chi-square tests or Fisher’s exact tests were performed as appropriate. The primary analysis was based on the primary outcome (endometrial thickness at 1 week post-surgery). Secondary outcome analyses were considered exploratory and were interpreted without global multiplicity adjustment beyond the prespecified Dunn–Bonferroni *post-hoc* comparisons. In addition, we conducted a subgroup analysis in the high-risk population (54 patients with a history of missed abortion, prior abortions, or mid-trimester pregnancy termination), comparing all outcome measures across the three groups. Linear regression analysis was then applied to further explore the relationship between these indicators and the treatment groups. Group 2 was used as the reference, as it represents the standard clinical treatment, and potential confounding factors were adjusted for in various models (the crude model was not adjusted; model 1 adjusted for age, education level, and marital status; model 2 adjusted for body mass index, smoking, and drinking; model 3 adjusted for miscarriages, day of menstruation, day of amenorrhea, history of special abortion, and history of uterine cavity surgery). Model selection was guided by clinical relevance and prior literature; covariates were chosen *a priori* rather than by stepwise selection.

Additionally, Kaplan–Meier (KM) analysis and log-rank tests were used to assess the effects of the three treatment regimens on the positive observational indicators. Although dichotomization of continuous variables may reduce statistical power, it was employed here to facilitate clinically interpretable time-to-event analysis, as KM analysis requires a binary event definition. Three parameters were dichotomized using population median values: (1) time to postoperative abdominal pain resolution, (2) endometrial thickness at 1 week post-surgery, and (3) postoperative vaginal bleeding time. Values below their respective medians were classified as positive events (i.e., whether postoperative abdominal pain resolved, whether favorable endometrial thickness was achieved, and whether postoperative vaginal bleeding ceased). For time to menstruation resumption, a threshold of 28 days (the typical menstrual cycle length of healthy individuals) was used, with ≤28 days considered a positive event (menstruation recovery). Similarly, for postoperative menstruation duration, 5 days was used as the threshold, with ≤5 days classified as a positive event (normal menstruation duration). Statistical significance was set at *p* < 0.05.

## 3 Results

### 3.1 Baseline of patients

The results of the baseline comparison indicated that there were no statistically significant differences in the age and clinical symptoms (education level, marital status, smoking, drinking, special abortion history, history of uterine surgery, times of miscarriages, menstruation, amenorrhea, body mass index, age) among the three groups of patients (Table 1, *p* > 0.05), suggesting that the groups were comparable at baseline.

**Table 1:** Baseline comparison of different groups of patients.

Variables	Total *n* = 380	Group 1 (*n* = 129)	Group 2 (*n* = 118)	Group 3 (*n* = 133)	*F*/*χ*^2^	*p*
Education level, *n* (%)						
Low	193 (50.789)	63 (48.837)	64 (54.237)	66 (49.624)	1.889	0.756
Middle	131 (34.474)	47 (36.434)	40 (33.898)	44 (33.083)		
High	56 (14.737)	19 (14.729)	14 (11.864)	23 (17.293)		
Marital status, *n* (%)						
Married	311 (81.842)	104 (80.620)	93 (78.814)	114 (85.714)	2.201	0.333
Other	69 (18.158)	25 (19.380)	25 (21.186)	19 (14.286)		
Smoking, *n* (%)						
No	355 (93.421)	116 (89.922)	106 (89.831)	133 (100.000)		
Yes	25 (6.579)	13 (10.078)	12 (10.169)	0 (0.000)		
Drinking, *n* (%)						
No	355 (93.421)	123 (95.349)	107 (90.678)	125 (93.985)	2.293	0.318
Yes	25 (6.579)	6 (4.651)	11 (9.322)	8 (6.015)		
Special abortion history, *n* (%)						
No	326 (85.789)	115 (89.147)	98 (83.051)	113 (84.962)	1.994	0.369
Yes	54 (14.211)	14 (10.853)	20 (16.949)	20 (15.038)		
History of uterine surgery, *n* (%)						
No	362 (95.263)	124 (96.124)	113 (95.763)	125 (93.985)	0.759	0.684
Yes	18 (4.737)	5 (3.876)	5 (4.237)	8 (6.015)		
Miscarriages, (times)	2.000 [2.000, 3.000]	2.000 [2.000, 3.000]	2.000 [2.000, 3.000]	2.000 [2.000, 3.000]	0.747	0.688
Menstruation, (day)	30.000 [29.000, 30.000]	30.000 [29.000, 30.000]	30.000 [29.000, 30.000]	30.000 [29.000, 30.000]	0.809	0.667
Amenorrhea, (day)	50.000 [46.000, 61.000]	50.000 [45.000, 60.000]	50.000 [46.000, 62.000]	51.000 [47.000, 64.000]	1.635	0.441
Body mass index, (kg/m^2^)	22.641 [20.868, 24.932]	22.719 [21.043, 24.741]	22.676 [20.701, 25.367]	22.513 [20.727, 24.737]	0.552	0.759
Age, (year)	33.000 [28.000, 37.000]	33.000 [28.000, 37.000]	33.000 [29.000, 37.000]	33.000 [28.000, 36.000]	0.699	0.705

Low education level includes junior high school, primary school, and secondary vocational, middle education level includes high school and junior college, and high education level includes undergraduate. Other marital status includes divorce and unmarried. Group 1: Honghua Ruyi Pills treatment. Group 2: Estradiol valerate + dydrogesterone tablets treatment. Group 3: Honghua Ruyi Pills + estradiol valerate + dydrogesterone tablets treatment.

### 3.2 Distribution of overall observational indicators

**Figure 1:**
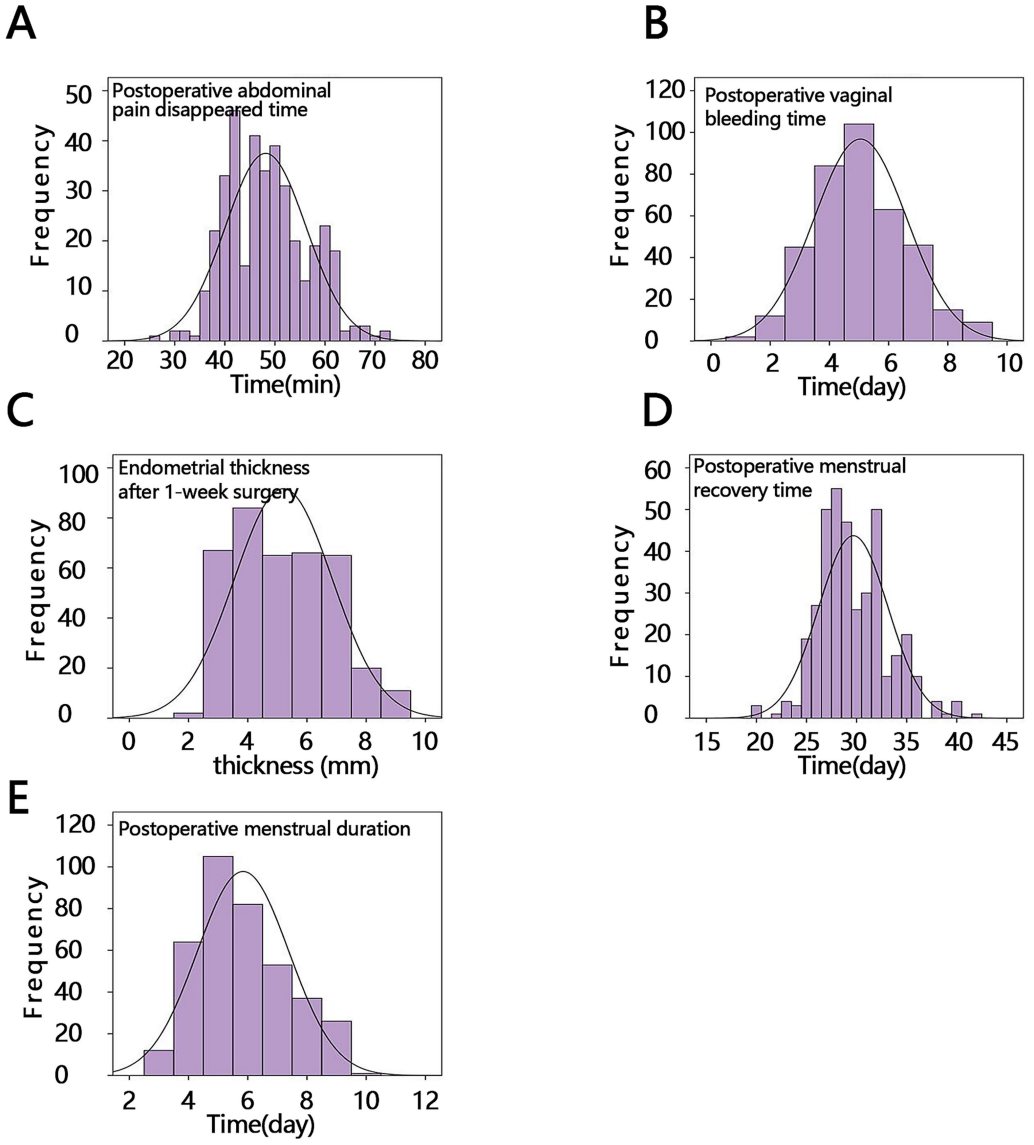
Histogram frequency distribution diagram of five postoperative outcome indicators in all patients. **(A)** Time to postoperative abdominal pain resolution. **(B)** Postoperative vaginal bleeding time. **(C)** Endometrial thickness at 1 week post-surgery. **(D)** Postoperative menstrual recovery time. **(E)** Postoperative menstrual duration.

To assess the postoperative recovery of all patients, we used frequency histograms to visually summarize the distributions of the five continuous observational indicators. The histograms showed that most observations clustered around the medians reported in Table 2, with mild skewness present for several variables. In line with the formal Shapiro–Wilk testing described in section 2.8, these visual displays were used descriptively rather than as evidence of normality. Figure 1 provides an overview of data dispersion and concentration, whereas the inferential comparisons were based on the non-parametric analyses reported below.

**Table 2:** Comparison of postoperative recovery indexes in different groups of patients.

Variables	Total *n* = 380	Group 1 (*n* = 129)	Group 2 (*n* = 118)	Group 3 (*n* = 133)	*F*/*χ*^2^	*p*
Time to postoperative abdominal pain resolution, (min)	48.000 [42.000, 53.000]	53.000 [49.000, 59.000]	48.000 [42.000, 52.000]	42.000 [40.000, 47.000]	84.401	<0.001
Postoperative vaginal bleeding time, (day)	5.000 [4.000, 6.000]	6.000 [5.000, 7.000]	5.000 [4.000, 6.000]	4.000 [3.000, 5.000]	130.121	<0.001
Time to menstruation resumption, (day)	29.000 [27.000, 32.000]	32.000 [29.000, 34.000]	29.000 [28.000, 31.000]	28.000 [26.000, 29.000]	63.832	<0.001
Postoperative menstrual duration, (day)	6.000 [5.000, 7.000]	7.000 [6.000, 8.000]	5.000 [5.000, 6.000]	5.000 [4.000, 6.000]	124.401	<0.001
Endometrial thickness at 1 week post-surgery, (mm)	5.000 [4.000, 7.000]	5.000 [4.000, 6.000]	5.000 [4.000, 6.000]	6.000 [4.000, 7.000]	12.216	0.002
Menstrual volume after menstruation, *n* (%)						
More	112 (29.474)	43 (33.333)	32 (27.119)	37 (27.820)	1.883	0.757
Normal	124 (32.632)	39 (30.233)	38 (32.203)	47 (35.338)		
Less	144 (37.895)	47 (36.434)	48 (40.678)	49 (36.842)		
Postoperative menstrual blood color, *n* (%)						
Dark reddish brownish	117 (30.789)	45 (34.884)	34 (28.814)	38 (28.571)	6.068	0.194
Black red	136 (35.789)	42 (32.558)	51 (43.220)	43 (32.331)		
Red	127 (33.421)	42 (32.558)	33 (27.966)	52 (39.098)		

Group 1: Honghua Ruyi Pills treatment. Group 2: Estradiol valerate + dydrogesterone tablets treatment. Group 3: Honghua Ruyi Pills + estradiol valerate + dydrogesterone tablets treatment.

### 3.3 Comparison of observational indicators among the three groups

The results of the Kruskal–Wallis test revealed significant statistical differences in time to postoperative abdominal pain resolution, postoperative vaginal bleeding time, postoperative menstrual recovery time, postoperative menstrual duration, and endometrial thickness at 1 week post-surgery among the three groups (*p* < 0.05, Table 2). However, there were no statistically significant differences in the distribution of menstrual volume after menstruation (across three levels) and postoperative menstrual blood color (three colors) between the groups (*p* > 0.05, Table 2). In the high-risk subgroup, Group 3 demonstrated significantly shorter time to postoperative abdominal pain resolution, postoperative vaginal bleeding duration, time to menstruation resumption, and postoperative menstrual duration compared with Groups 1 and 2 (*p* < 0.05), while endometrial thickness at 1 week post-surgery did not differ significantly among the three groups (*p* = 0.117). No statistically significant differences were observed among the three groups in menstrual volume (*p* = 0.118) or postoperative menstrual blood color (*p* = 0.464) (Supplementary Table S1).

**Figure 2:**
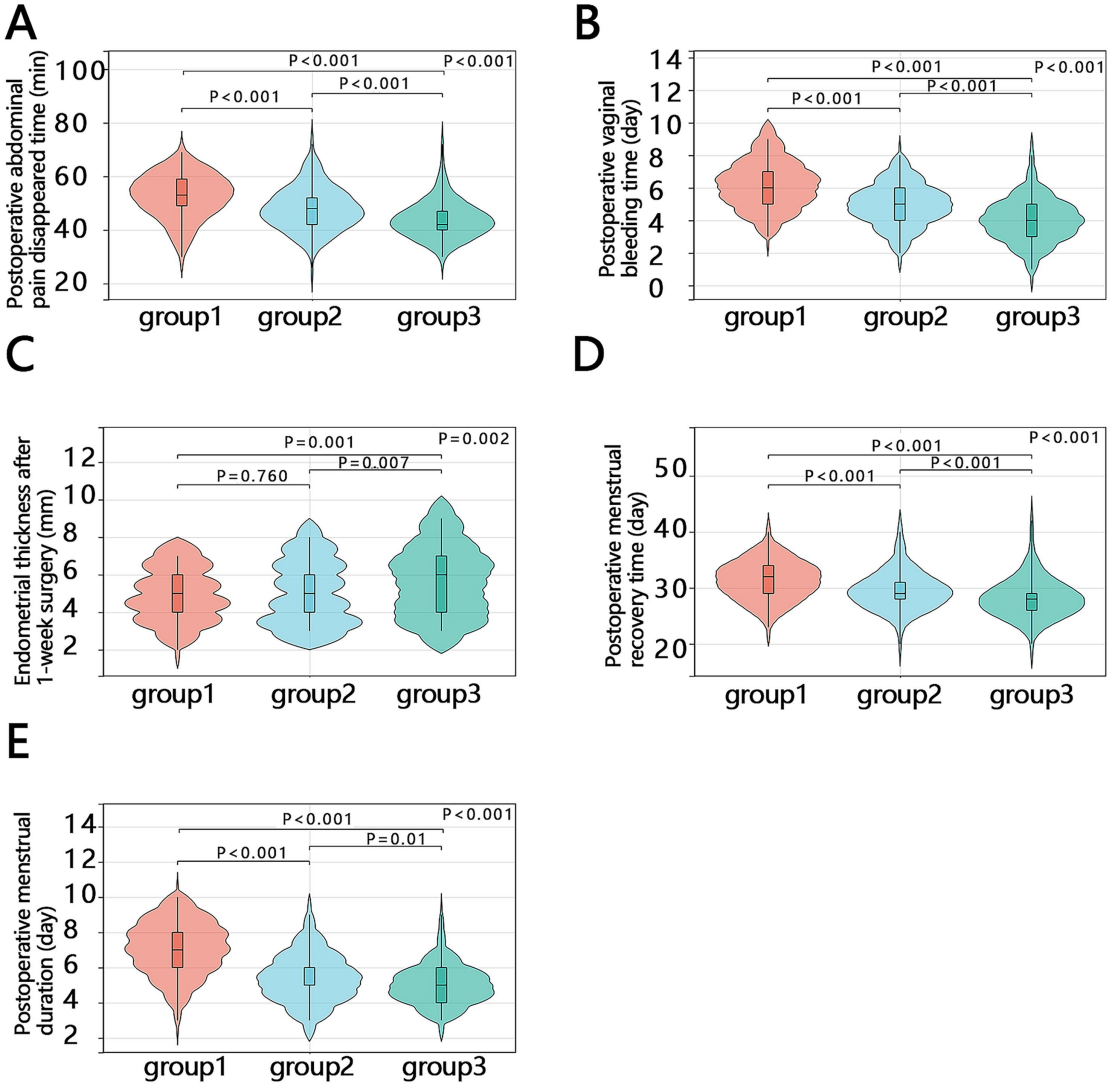
*Post-hoc* comparisons of five postoperative outcome indicators among the three groups. **(A)** Time to postoperative abdominal pain resolution. **(B)** Postoperative vaginal bleeding time. **(C)** Endometrial thickness at 1 week post-surgery. **(D)** Time to menstruation resumption. **(E)** Postoperative menstrual duration. Group 1: Honghua Ruyi Pills treatment. Group 2: Estradiol valerate + dydrogesterone tablets treatment. Group 3: Honghua Ruyi Pills + estradiol valerate + dydrogesterone tablets treatment.

To more accurately compare the differences between the three groups for these indicators, Dunn–Bonferroni *post-hoc* comparisons were further conducted after the overall Kruskal–Wallis tests. Using the Bonferroni-adjusted threshold of *p* < 0.0167, Group 1 showed longer time to postoperative abdominal pain resolution, postoperative bleeding cessation, time to menstruation resumption, and postoperative menstrual duration than Groups 2 and 3. In addition, Group 2 had longer time to postoperative abdominal pain resolution than Group 3. For endometrial thickness at 1 week post-surgery, Group 3 showed greater values than Groups 1 and 2, whereas no significant difference was observed between Groups 1 and 2 (Figure 2).

### 3.4 Relationship between treatment methods and primary observational indicators

The above results indicate that the three treatment methods significantly affect the five observational indicators. However, the precise relationships between them are unclear. Therefore, we further performed linear regression to explore the relationships between the treatment methods and the five observational indicators. Since Group 2 represents the standard clinical treatment, it was used as the reference group. In the crude model, we found that treatment method in Group 3 was significantly negatively correlated with the postoperative abdominal pain time, vaginal bleeding time, menstrual recovery time, and menstrual duration. But it was positively related to the endometrial thickness at 1 week post-surgery. When adjusting for other confounding factors, their relationship remained significant (all *p* < 0.05, Supplementary Table S2).

**Figure 3:**
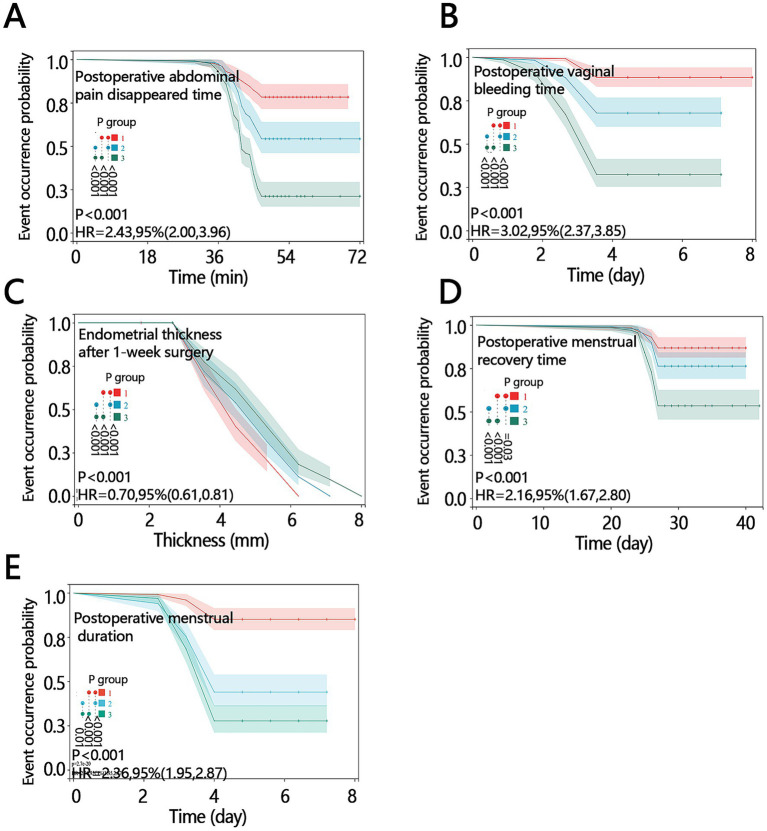
KM analysis of five postoperative outcome indicators **(A–E)**. **(A)** Time to postoperative abdominal pain resolution. **(B)** Postoperative vaginal bleeding time. **(C)** Endometrial thickness at 1 week post-surgery. **(D)** Time to menstruation resumption. **(E)** Postoperative menstrual duration. Group1: Honghua Ruyi Pills treatment. Group 2: Estradiol valerate + dydrogesterone tablets treatment. Group 3: Honghua Ruyi Pills + Estradiol valerate + dydrogesterone tablets treatment.

Additionally, KM analysis was used to evaluate the effects of different treatments on the five prespecified postoperative recovery indicators. The log-rank tests showed significant between-group differences for abdominal pain resolution, bleeding cessation, endometrial thickness at 1 week post-surgery, time to menstruation resumption, and postoperative menstrual duration (Figure 3, *p* < 0.05). Patients in Group 3 tended to achieve favorable postoperative recovery milestones earlier than those in the other groups. These time-to-event findings should be interpreted as supportive exploratory analyses of the primary and secondary outcome results.

### 3.5 Comparison of adverse symptoms and reactions among the groups

To compare the safety of the three treatment regimens, we recorded the frequency of adverse symptoms and reactions in each group. The results indicated that adverse reactions occurred in all three groups, including postoperative complications, gastrointestinal discomfort, breast tenderness, headaches, and other symptoms. However, there were no significant differences in the frequency of adverse events among the three groups (*p* > 0.05, Table 3).

**Table 3:** Comparison of postoperative adverse reactions of different medication methods.

Variables	Total (*n* = 380)	Group 1 (*n* = 129)	Group 2 (*n* = 118)	Group 3 (*n* = 133)	*χ* ^2^	*p*
Postoperative complication, *n* (%)						
No	312 (82.105)	107 (82.946)	92 (77.966)	113 (84.962)	2.177	0.337
Yes	68 (17.895)	22 (17.054)	26 (22.034)	20 (15.038)		
Stomach discomfort, *n* (%)						
No	365 (96.053)	128 (99.225)	111 (94.068)	126 (94.737)	5.257	0.072
Yes	15 (3.947)	1 (0.775)	7 (5.932)	7 (5.263)		
Breast distending pain, *n* (%)						
No	369 (97.105)	125 (96.899)	114 (96.610)	130 (97.744)	0.316	0.854
Yes	11 (2.895)	4 (3.101)	4 (3.390)	3 (2.256)		
Headache, *n* (%)						
No	366 (96.316)	126 (97.674)	113 (95.763)	127 (95.489)	1.029	0.598
Yes	14 (3.684)	3 (2.326)	5 (4.237)	6 (4.511)		
Diarrhea, *n* (%)						
No	358 (94.211)	124 (96.124)	108 (91.525)	126 (94.737)	2.493	0.287
Yes	22 (5.789)	5 (3.876)	10 (8.475)	7 (5.263)		
Intrauterine adhesions, *n* (%)						
No	378 (99.474)	128 (99.225)	118 (100.000)	132 (99.248)		
Yes	2 (0.526)	1 (0.775)	0 (0.000)	1 (0.752)		
Residual uterine cavity, *n* (%)						
No	377 (99.211)	127(98.450)	117(99.153)	133(100.000)		
Yes	3 (0.789)	2(1.550)	1(0.847)	0(0.000)		
Pelvic inflammatory disease, *n* (%)						
No	379 (99.737)	129 (100.000)	117 (99.153)	133 (100.000)		
Yes	1 (0.263)	0 (0.000)	1 (0.847)	0 (0.000)		
Periodic abdominal pain, *n* (%)						
No	369 (97.105)	124 (96.124)	115 (97.458)	130 (97.744)	0.687	0.709
Yes	11 (2.895)	5 (3.876)	3 (2.542)	3 (2.256)		

The frequency of intrauterine adhesions, residual uterine cavity, and pelvic inflammatory disease in some groups is 0, so the chi-square test cannot be used for comparison. Group 1: Honghua Ruyi Pills treatment. Group 2: Estradiol valerate + dydrogesterone tablets treatment. Group 3: Honghua Ruyi Pills + estradiol valerate + dydrogesterone tablets treatment.

## 4 Discussion

This study primarily found that the combination of Honghua Ruyi Pills and estradiol valerate and dydrogesterone tablets had a positive effect on postoperative recovery following artificial abortion. Specifically, the combination of Honghua Ruyi Pills and estradiol valerate and dydrogesterone tablets significantly shortened the duration of postoperative abdominal pain and vaginal bleeding, and facilitated the endometrial restoration, allowing patients to return to a normal physiological cycle more quickly. Post-abortion surgical trauma induces progressive thinning of the functional endometrial layer, leading to a marked decline in endometrial volume and reduction in thickness. These structural alterations may impair ovarian function and disrupt hormonal cyclicity, thereby increasing susceptibility to potential gynecological complications (26, 27). Additionally, induced abortion may compromise the integrity of the endometrial basal layer, damaging its vascular supply. The reduction in diameter of endometrial arteries impairs microcirculation within the basal layer, further disrupting normal blood perfusion. These hemodynamic and structural abnormalities impede endometrial regeneration, thereby prolonging the restoration of regular menstrual cyclicity (28).

Estrogen-progestin medications can promote the repair of the endometrium through artificial cycle therapy. Among them, estradiol valerate tablets and dydrogesterone are commonly used in clinical practice for uterine repair. Estradiol valerate tablets are a natural estrogen drug, with estradiol valerate as the main ingredient. After being absorbed and metabolized by the body, it produces valeric acid and the active estrogen estradiol. Estradiol binds with estrogen receptors and stimulates rapid division of endometrial cells, thus promoting endometrial proliferation (29). It has a reparative effect on damaged endometrial cells, and when the repair process of the endometrium is effectively promoted, it reduces the formation of tissue scars. Dydrogesterone tablets can reduce cervical mucus secretion, thereby decreasing or preventing the upward invasion of bacteria, which in turn reduces the risk of intrauterine adhesions (30, 31). Dydrogesterone is a progesterone isomer that is commonly used to treat diseases caused by endogenous progesterone deficiency, such as dysfunctional uterine bleeding and menstrual cycle disorders. Dydrogesterone has a high affinity and inhibits the secretion of pituitary gonadotropins, protecting the endometrium and preventing excessive endometrial proliferation. It effectively accelerates the shedding of the endometrium and facilitates endometrial shedding bleeding (32, 33). The choice of 1 mg estradiol valerate, rather than higher doses (2–5 mg) used in studies of severe intrauterine adhesions, reflects the relatively intact endometrial basal layer and estrogen receptor expression in our population following artificial abortion. At this dose, serum estradiol reaches mid-follicular phase levels, sufficient to drive endometrial proliferation without supraphysiological stimulation (34). Dydrogesterone was introduced on day 11 to mimic the natural luteal transition: after approximately 10 days of estrogenic priming, progestogen addition promotes the shift from proliferative to secretory endometrium (35). Premature progestogen exposure would antagonize estrogen-driven proliferation, while delayed introduction risks uncontrolled growth; day 11 therefore represents a physiologically appropriate and clinically pragmatic time point.

In traditional Chinese medicine, patients undergoing artificial abortion often experience damage to kidney energy, imbalance in Qi and blood, and residual blood stasis. As a result, these patients typically present with symptoms of kidney deficiency and blood stasis, leading to poor circulation of Qi and blood post-surgery, which increases the risk of complications such as intrauterine adhesions. Honghua Ruyi Pills, derived from a classic Tibetan medicine formula, have properties of invigorating blood circulation, dissipating blood stasis, and regulating menstruation while relieving pain. It has been widely used throughout history to treat various gynecological conditions, promoting the flow of Qi and blood to facilitate recovery (21). The combination of *Carthamus tinctorius* L. and *Crocus sativus* L. exerts dual regulatory effects on hemorheological dysfunction by improving microcirculatory parameters and resolving pathological stasis. Whereas *Inularacemosa Hook. f.* and *Terminalia chebula Retz* enhance the flow of Qi and blood. Additionally, *Corydalis hendersonii Hemsl.* helps to clear blood heat and alleviate blood stasis, while *Coriandrum sativum* and *Ursi fellis pulvis* work to eliminate both stagnation and heat. Furthermore, *Cinnamomum cassia Presl* can balance the overall medicinal properties of the formula, preventing excessive coldness that could adversely affect the stomach (36, 37). This complete formula promotes blood circulation, eliminates stasis, unblocks meridians, and regulates Qi and blood, thereby aiding in endometrial repair and the restoration of a normal menstrual cycle.

The observed reduction in postoperative vaginal bleeding duration in Group 3, despite the use of Honghua Ruyi Pills—agents that promote Huoxue Huayu (blood invigoration and stasis resolution)—may appear counterintuitive given conventional concerns about prolonged bleeding with blood-activating botanicals. Two complementary mechanisms may account for this finding. First, the blood-invigorating constituents of the formula, particularly *Carthamus tinctorius* L. and *Crocus sativus* L., are known to enhance uterine smooth muscle contractility (38–40), which facilitates the timely expulsion of intrauterine remnants such as residual decidua and clots; prompt clearance of these bleeding sources may itself shorten the overall bleeding duration. Second, as detailed below, suppression of TNF-mediated inflammatory signaling by Honghua Ruyi Pills is expected to attenuate postoperative uterine inflammation, accelerating endometrial microvascular repair and thereby hastening hemostasis (41). Collectively, the hemostatic benefit observed in Group 3 likely reflects an acceleration of physiological uterine wound resolution rather than direct hemorrhagic suppression—a mechanistic distinction with important implications for clinical interpretation of blood-invigorating therapies in the postoperative setting.

Moreover, network pharmacology analysis has demonstrated that key target genes of Honghua Ruyi Pills include ESR1, which is predominantly distributed in estrogen-responsive tissues. ESR1 encodes the estrogen receptor that plays a critical role in modulating estrogen levels (42). The therapeutic effects of Honghua Ruyi Pills on endometrial repair may stem from its regulatory influence on ESR1 expression. Additionally, TNF has been identified as another pivotal target of Honghua Ruyi Pills. Notably, TNF serves as a biomarker for peritoneal adhesion and participates in inflammatory cascades. Elevated TNF levels are closely associated with inflammatory conditions (41). Considering these findings, we postulate that Honghua Ruyi Pills may attenuate post-abortion inflammatory responses induced by surgical trauma, thereby facilitating endometrial and uterine functional recovery.

Importantly, the present study focused primarily on short-term surrogate outcomes such as pain duration, bleeding duration, and endometrial thickness at 1 week post-surgery, without assessing clinically meaningful reproductive endpoints. Specifically, pregnancy rate, hysteroscopy-confirmed intrauterine adhesion formation, and live birth rate were not evaluated. The absence of these endpoints limits the translational impact of our findings, because improvements in surrogate markers such as endometrial thickness may not reliably predict long-term reproductive outcomes (23). Future studies should incorporate these clinically relevant endpoints to establish the definitive clinical benefit of the combination therapy.

Compared to previous studies (43), this randomized controlled trial provides additional short-term evidence that combining Honghua Ruyi Pills with estradiol valerate and dydrogesterone tablets may facilitate postoperative endometrial recovery and menstrual restoration after artificial abortion. However, given the short follow-up duration, the reliance on surrogate outcomes, and the absence of key reproductive endpoints, these findings should be interpreted as preliminary rather than definitive. At present, the results support further validation in multicenter studies rather than immediate broad generalization to routine clinical practice.

However, several limitations of this study should be acknowledged. First, clinical surveillance was confined to ultrasonographic assessment of endometrial thickness and uterine cavity morphology. Vital signs, hemoglobin levels for objective bleeding quantification, and hepatic/renal function tests were not systematically monitored as predefined outcomes, which may limit the comprehensive evaluation of safety, particularly given the cinnabar content in Honghua Ruyi Pills. Second, ultrasound was performed only at 1 week post-surgery, when residual edema or bleeding may still influence the measurements. Additional assessments at day 21 or before the first post-treatment menstruation, including endometrial pattern and sub-endometrial blood-flow parameters, would provide more robust recovery data. Third, the 21-day observation period is insufficient to determine whether the intervention prevents longer-term intrauterine adhesion formation, as adhesions may develop over months. Moreover, clinically meaningful reproductive endpoints, including hysteroscopy-confirmed intrauterine adhesion rate, pregnancy rate, and live birth rate, were not assessed. Follow-up at 3 to 6 months incorporating repeat ultrasonography, hysteroscopy, and evaluation of reproductive outcomes is needed to confirm sustained efficacy and strengthen the translational value of the findings. Fourth, as a single-center study conducted within a specific integrative treatment context combining traditional Chinese medicine (Tibetan medicine) with conventional hormonal therapy, the generalizability of the results to broader reproductive medicine practice across different populations and clinical settings remains uncertain. Future multicenter studies with diverse patient populations, extended follow-up, comprehensive safety monitoring, and inclusion of definitive reproductive endpoints are therefore warranted to address these limitations.

## 5 Conclusion

This single-center, randomized, double-blind trial provides preliminary evidence that the combination of Honghua Ruyi Pills with estradiol valerate and dydrogesterone tablets may promote short-term endometrial recovery and menstrual cycle restoration in patients after artificial abortion more effectively than either treatment alone. However, due to the reliance on surrogate endpoints, the short observation period, and the single-center design, these findings should be interpreted as hypothesis-generating. Multicenter trials with longer follow-up periods and clinically relevant reproductive endpoints, including pregnancy rate, intrauterine adhesion formation, and live birth rate, are needed to confirm the clinical benefit of this combination therapy.

## Data Availability

The raw data supporting the conclusions of this article will be made available by the authors, without undue reservation.
