# Supplementary material for: The multiple positive effects of Honghua Ruyi Pills combined with estradiol valerate and dydrogesterone tablets on postoperative recovery in women after artificial abortion
Source: Front Med (Lausanne). 2026 Apr 22;13:1778706. doi: 10.3389/fmed.2026.1778706 (PMC13144064; doi:10.3389/fmed.2026.1778706)
Supplement: Supplementary file 1 [file Table_1.DOCX]

| **Table S1 Comparison of postoperative outcomes among three treatment groups in the high-risk subgroup** | | | | |
| --- | --- | --- | --- | --- |
| variables | group 1 (n = 14) | group 2 (n = 20) | group 3 (n = 20) | P |
| time to postoperative abdominal pain resolution (min) | 53.5 (53.50 ± 7.56) | 49.0 (47.95 ± 6.97) | 43.5 (42.75 ± 5.21) | < 0.001 |
| postoperative vaginal bleeding time (days) | 6.0 (5.71 ± 1.54) | 5.0 (5.20 ± 1.20) | 4.0 (3.90 ± 1.02) | < 0.001 |
| time to menstruation resumption (days) | 32.0 (30.71 ± 4.01) | 31.0 (30.80 ± 2.17) | 28.0 (27.60 ± 3.17) | 0.003 |
| postoperative menstrual duration (days) | 7.0 (7.14 ± 1.10) | 6.0 (5.85 ± 1.31) | 5.0 (4.85 ± 0.75) | < 0.001 |
| endometrial thickness at 1 week post-surgery (mm) | 5.0 (5.29 ± 1.27) | 4.0 (4.95 ± 1.93) | 6.0 (6.10 ± 1.68) | 0.117 |
| menstrual volume after menstruation | | | | 0.118 |
| less | 6 (42.9%) | 3 (15.0%) | 6 (30.0%) |  |
| normal | 3 (21.4%) | 5 (25.0%) | 9 (45.0%) |  |
| more | 5 (35.7%) | 12 (60.0%) | 5 (25.0%) |  |
| postoperative menstrual blood color | | | | 0.464 |
| black red | 4 (28.6%) | 6 (30.0%) | 5 (25.0%) |  |
| red | 8 (57.1%) | 10 (50.0%) | 7 (35.0%) |  |
| dark reddish brownish | 2 (14.3%) | 4 (20.0%) | 8 (40.0%) |  |

Note: group1: Honghua Ruyi pills treatment; group2: estradiol valerate + dydrogesterone tablets treatment; group3: Honghua Ruyi pills + estradiol valerate + dydrogesterone tablets treatment
