# Supplementary material for: The multiple positive effects of Honghua Ruyi Pills combined with estradiol valerate and dydrogesterone tablets on postoperative recovery in women after artificial abortion
Source: Front Med (Lausanne). 2026 Apr 22;13:1778706. doi: 10.3389/fmed.2026.1778706 (PMC13144064; doi:10.3389/fmed.2026.1778706)
Supplement: Supplementary file 2 [file Table_2.DOCX]

| **Table S2 The relationship between different medication methods and postoperative recovery indexes of patients** | | | | | | | | |
| --- | --- | --- | --- | --- | --- | --- | --- | --- |
|  | crude model | | model1 | | model2 | | model3 |  |
| variables | β[95%CI] | P | β[95%CI] | P | β[95%CI] | P | β[95%CI] | P |
| time to postoperative abdominal pain resolution |  |  |  |  |  |  |  |  |
| group2 | ref | ref | ref | ref | ref | ref | ref | ref |
| group1 | 4.600[2.802,6.504] | <0.001 | 4.701[2.903,6.532] | <0.001 | 4.721[2.923,6.533] | <0.001 | 4.606[2.853,6.434] | <0.001 |
| group3 | -4.401[-5.805,-2.204] | <0.001 | -3.801[-5.603,-2.034] | <0.001 | -4.101[-6.063,-2.342] | <0.001 | -3.802[-5.612,-2.032] | <0.001 |
| postoperative vaginal bleeding time |  |  |  |  |  |  |  |  |
| group2 | ref | ref | ref | ref | ref | ref | ref | ref |
| group1 | 1.201[0.904,1.603] | <0.001 | 1.221[0.955,1.623] | <0.001 | 1.221[0.916,1.506] | <0.001 | 1.205[0.912,1.623] | <0.001 |
| group3 | -0.923[-1.357,-0.621] | <0.001 | -1.012[-1.323,-0.634] | <0.001 | -0.923[-1.305,-0.623] | <0.001 | -1.036[-1.323,-0.744] | <0.001 |
| time to menstruation resumption |  |  |  |  |  |  |  |  |
| group2 | ref | ref | ref | ref | ref | ref | ref | ref |
| group1 | 1.801[1.045,2.623] | <0.001 | 1.802[1.023,2.632] | <0.001 | 1.805[0.923,2.632] | <0.001 | 1.806[1.034,2.623] | <0.001 |
| group3 | -1.302[-2.177,-0.534] | <0.001 | -1.401[-2.223,-0.634] | <0.001 | -1.327[-2.123,-0.531] | <0.001 | -1.427[-2.221,-0.623] | <0.001 |
| postoperative menstrual duration |  |  |  |  |  |  |  |  |
| group2 | ref | ref | ref | ref | ref | ref | ref | ref |
| group1 | 1.721[1.406,2.046] | <0.001 | 1.702[1.323,2.045] | <0.001 | 1.702[1.313,2.023] | <0.001 | 1.706[1.432,2.043] | <0.001 |
| group3 | -0.421[-0.705,-0.107] | <0.001 | -0.401[-0.734,-0.143] | <0.001 | -0.403[-0.725,-0.102] | <0.001 | -0.447[-0.734,-0.132] | <0.001 |
| endometrial thickness at 1 week post-surgery |  |  |  |  |  |  |  |  |
| group2 | ref | ref | ref | ref | ref | ref | ref | ref |
| group1 | -0.112[-0.503,0.302] | 0.501 | -0.109[-0.503,0.323] | 0.646 | -0.202[-0.645,0.356] | 0.501 | -0.125[-0.504,0.343] | 0.602 |
| group3 | 0.621[0.276,1.023] | <0.001 | 0.601[0.246,1.109] | <0.001 | 0.601[0.223,1.090] | <0.001 | 0.621[0.223,1.133] | <0.001 |

Note: crude model: without adjustment

model1: adjusting for age, education level, marital status

model2: adjusting for body mass index, smoking, drinking

model3: adjusting for times of miscarriages, day of menstruation, day of amenorrhea, history of special abortion, history of uterine cavity surgery

group1: Honghua Ruyi pills treatment; group2: estradiol valerate + dydrogesterone tablets treatment; group3: Honghua Ruyi pills + estradiol valerate + dydrogesterone tablets treatment
